# Studies on the Interaction Between the Functional Monomer 4-Methacryloxyethyl Trimellitic Anhydride and Hydroxyapatite and Stability of the Obtained Hybrids

**DOI:** 10.3390/ma18081689

**Published:** 2025-04-08

**Authors:** Vasil Kalchinov, Kostadinka Sezanova, Pavletta Shestakova, Sevda Yantcheva, Radosveta Vasileva, Diana Rabadjieva

**Affiliations:** 1 Faculty of Dental Medicine, Medical University, 1 G. Sofiiski Str., 1431 Sofia, Bulgaria; sevda.yancheva@gmail.com (S.Y.); eitenet@abv.bg (R.V.); 2 Institute of General and Inorganic Chemistry, Bulgarian Academy of Sciences, Acad. G. Bonchev Str., bl. 11, 1113 Sofia, Bulgaria; ksezanova@svr.igic.bas.bg; 3 Institute of Organic Chemistry with Centre of Phytochemistry, Bulgarian Academy of Sciences, Acad. G. Bonchev Str., bl. 9, 1113 Sofia, Bulgaria; pavletta.shestakova@orgchm.bas.bg

**Keywords:** 4-methacryloxyethyl trimellitic anhydride, hydroxyapatite, adhesive, solid-state NMR, kinetics

## Abstract

Minimizing the risk of secondary caries in dentistry is achieved by using adhesive systems that provide a strong bond between the natural hard tissue and the restorative material. Evaluating the effectiveness of these systems requires studying both their interaction with dentin and enamel and their behavior in environments with varying acidity. In this work, the interaction of a reactive monomer, 4-methacryloxyethyl trimellitic anhydride (4-META), used in adhesive systems with both dentin-like hydroxyapatite (HA) and hydroxyapatite ceramics, was investigated. Kinetic studies showed that under experimental conditions, 4-META was hydrolyzed and amorphized. Dentin-like HA possessed greater adsorption capacity to 4-META than ceramic HA. Immersion of HA into a solution of 4-META led to formation of an acidic calcium phosphate phase over time in both systems. Studies on the solubility of the synthetic nanosized hydroxyapatite and its derivative with 4-META in 0.1 mol/L lactic acid, also containing CaCl_2_, Na_2_HPO_4_, and NaF (pH 4.5), and in distilled water (pH 6.3) indicated the occurrence of dissolution, complexation, and crystallization processes, causing changes in the liquid and solid phases. The total Ca^2+^ concentration upon dissolution of hybrid HA-4-META in a lactic acid solution was three times lower than the total Ca^2+^ concentration upon dissolution of pure HA. This suggested that 4-META-treated dentin-like surfaces demonstrate greater resistance to dissolution in acidic environments compared to untreated surfaces, highlighting the potential for these hybrids in dental applications.

## 1. Introduction

Secondary caries is a serious problem in operative dentistry. It can form in dental amalgams, glass ionomer cement (GIC), and composite fillings [1,2], leading to complications such as damage to the dental pulp and periodontium [3,4]. The adhesive systems used in dental restorations are essential for minimizing the risk of secondary caries by establishing a strong and stable bond between the restoration and the tooth structure. Dental adhesives typically contain reactive monomers possessing hydrophilic and hydrophobic groups. The hydrophilic groups bond to enamel, especially to wet dentin, while the hydrophobic groups bond to the composite restorative material [5,6]. Typical modern representatives of this type of material are dipentaerythritol pentaacrylate phosphoric acid ester (PENTA), 4-methacryloxyethyl trimellitate (4-MET) or its anhydride form (4-META), methacryloyloxydecyl dihydrogen phosphate (10-MDP), 2-methacryloyloxyethyl phenyl hydrogen phosphate (phenyl-P) [6,7], etc. Although it was initially believed that the micromechanical bond was the most important for adhesion [8], later studies have shown that the formation of a hybridization zone and chemical interaction provide better sealing and prevent microleakage in composite fillings. Acidic monomers, such as those mentioned above, can form a strong ionic bond with Ca^2+^ cations from the hydroxyapatite crystals of enamel or dentin, create stable salts that are highly insoluble in water, and some of them, such as 10-MDP, 4-META or 4-MET, can also interact stably with the collagen matrix of dentin [9,10,11,12,13].

4-META (C_15_H_12_O_7_, CAS 70293-55-9) and 4-MET (C_15_H_14_O_8_, CAS 68183-31-3) are particularly advantageous in adhesive dentistry because they combine chemical bonding ability, hydrophilicity, and flexibility. Their structures are similar, containing a methacryloxy group (-CH_2_=C(CH_3_)COO-) covalently linked to a mellitic moiety via a dimethylene bridge. In the case of 4-META, the mellitic entity is a mellitic anhydride, while for 4-MET, the moiety involved is a mellitic acid residue.

Fujisawa and Ito [14] found that in aqueous media, 4-META is rapidly converted to 4-MET, whose carboxylate groups interact with calcium cations of brushite (CaHPO_4_·2H_2_O). Using X-ray photoelectron spectroscopy and calcium phthalate as a model of 4-META, Ohno et al. [15] suggested that Ca of the bovine enamel and the carboxylic group of 4-META were chelate-bonded at the resin–enamel interface. Nagakane et al. [16] and Yoshida et al. [9] studied the interaction of 4-MET with calcium from synthetic hydroxyapatite (Ca_5_(PO_4_)_3_OH, HA). They also confirmed the formation of a chemical bond between HA and the monomer.

Synthetic hydroxyapatite substrates are usually used to model hard dental tissues. Enamel and dentin are the two mineralized layers of the tooth subjected to intense demineralization processes resulting from the fermentation of food carbohydrates and leading to the formation of caries. They are natural composites built up of an inorganic component, “biological apatite”, and an organic component, most commonly collagen. The “biological apatite” in both structures is nanoscale calcium hydroxyapatite, but in dentin, the size of apatite particles and its crystallinity are smaller than that of enamel. The hydroxyapatite crystals in enamel are uniformly oriented and regularly oriented, which determines their unique density and strength, while in dentin they are not so correctly oriented, and when they are arranged, they form microscopic canals called dental tubules, in which cells are located, allowing their regeneration [17].

Despite the accumulated research data, studies on the behavior and especially on the degradation chemistry and solubility of the reactive monomers in the adhesive systems and covered with hard tissues are insufficient. This information is important for assessing the long-term clinical effectiveness of adhesive systems.

This work aimed to study both the interaction of the functional acidic monomer 4-META with hydroxyapatite (HA) particles and the stability of the resulting hybrids in environments with different pH to clarify the influence of pH in the oral cavity on the strength of the adhesive bond. To achieve the set goal, hydroxyapatite with two crystallite sizes and accordingly different values of the specific surface area were obtained as approximate models of enamel and dentin. Changes in the solid phases with time during the adsorption of a water–alcohol solution of 4-META on the hydroxyapatite surface were monitored using solid-state nuclear magnetic resonance analysis. The solubility of the synthetic nanosized hydroxyapatite and its derivative with 4-META in 0.1 mol/L lactic with pH 4.5 and in distilled water with pH 6.3 were further investigated. A lactic acid solution mimics the conditions in the oral cavity during the bactericidal breakdown of sugars, while the pH of distilled water is close to that of natural healthy saliva. The obtained results are discussed from the perspective of the difference in solubility and phase changes with time of pure hydroxyapatite, as a representative of hard dental tissue, and one treated with 4-META.

## 2. Materials and Methods

### 2.1. Synthesis of Hydroxyapatite

The continuous co-precipitation method was applied to synthesize hydroxyapatite. A 0.6 mol/L solution of (NH_4_)_2_HPO_4_ (Merck, Darmstadt, Germany, p.a.) was added at a rate of 5 mL/min to a 1 mol/L solution of Ca(NO_3_)_2_·4H_2_O (Merck, Darmstadt, Germany, p.a.) at a Ca/P ratio of 1.67 and constant pH 11 under intensive stirring at room temperature. In addition, a 25% NH_4_OH (Honeywell, Charlotte, NC, USA, p.a.) solution was used to adjust pH value to 11.

The suspension was matured for 1.5 h under stirring and another 20 h at rest. Then, the solid phase was separated by centrifugation (4000 rpm, 10 min), rinsed with water, and again centrifuged. The washed powder was dried for 24 h at 65 °C. This material is hereinafter referred to as HA65. Part of HA65 was sintered at 1000 °C and is hereinafter referred to as HA1000.

### 2.2. Interaction of the Functional Acidic Monomer 4-META with Hydroxyapatite

The obtained HA65 and HA1000 powders were ground individually for half an hour in a planetary ball mill at 500 rpm, followed by sieving through a sieve with an opening size of 0.16 mm. A fraction with a particle size below 0.16 mm was used in the experimental studies.

A mixed solution of 4-META (Sigma Aldrich, St. Louis, MO, USA), ethanol (Honeywell, Charlotte, NC, USA), and distilled deionized water in a ratio of 4-META:EtOH:H_2_O of 15:45:40 wt% was prepared [9]. Ten grams of the freshly prepared solution (pH 2) was placed in contact with two grams of HA65 or HA1000 at room temperature and constant stirring at a speed of 100 rpm. At given times (15 min, 1 h, 6 h, 24 h, and 14 days), the suspensions were centrifuged and the precipitate was washed three times with ethanol by centrifugation at 4000 rpm for 5 min. The final precipitates were air-dried for 72 h and are referred to as (HA65-4META)_t_ and (HA1000-4META)_t_, respectively, where “t” is the contact time—15 min, 1 h, 6 h, and 24 h.

### 2.3. Stability of the Hybrids in Environments with Different pH

Only (HA65-4META)_24h_ was used for the studies because of the highest monomer content in it. The experiments were performed under static conditions. The material (0.25 g) was placed in contact with 15 mL of distilled water (pH 6.3) or a solution of 0.1 mol/L lactic acid (L-(+)-lactic acid, Sigma-Aldrich, Overijse, Belgium) (pH 4.5) also containing 2.2 mmol/L CaCl_2_·2H_2_O (Sigma-Aldrich, A.R., St. Louis, MO, USA), 2.2 mmol/L Na_2_HPO_4_ (Merck, Darmstadt, Germany), and 0.005 mmol/L NaF (Merck, Darmstadt, Germany). The acidic solution has been used elsewhere for artificial demineralization of tooth enamel [18].

In the liquid phase, the change in the concentration of free Ca^2+^ ions and pH with time was monitored using a polymer membrane calcium ion-selective electrode (Methrom AG, Herisau, Switzerland) and combined pH electrode (iConneet, Methrom AG, Herisau, Switzerland), both connected to device for automatic titration Titrando 907 (Methrom AG, Switzerland). Measurements were made in situ during the first 24 h. A calibration standard curve for calculating free Ca^2+^ ion concentrations was generated by a measurement of the electrical potential of the standard solutions of Ca(NO_3_)_2_ in distilled water. The concentrations of the standard solutions were within the concentration range of the Ca^2+^ ions under the experimental conditions.

### 2.4. Characterization

#### 2.4.1. Chemical Analysis

Complexometric analysis using EDTA and the indicator eriochrome black T at pH 10 was used to determine the total concentration of Ca^2+^ ions in the liquid and solid phases, with the solids having been pre-dissolved in HNO_3_ (Merck, Darmstadt, Germany). A NOVA 60 spectrophotometer and Merck Spectroquant test kits were used to determine the concentrations of PO_4_^3−^ ions.

Ten parallel independent measurements were performed for each assay. The accuracy of the results is expressed by the standard deviation value. The accuracy of the Ca/P ratio was calculated using Equation (1):

(1) $$SR=\sqrt{\frac{s_{Ca}^{2}}{C_{Ca}}}+\sqrt{\frac{s_{P}^{2}}{C_{P}}}$$

where:

*SR* is the standard deviation of the Ca/P molar ratio;

*s_Ca_*, *s_p_*—standard deviation of Ca and P measurements, respectively;

*C_Ca_*, *C_P_*—average value of measurements of concentration of Ca and P in mmol/L.

#### 2.4.2. Powder X-Ray Diffraction (XRD) Analysis

A Bruker D8 Advance diffractometer with CuK-α radiation (λ = 1.5406 Å) and a LynxEye detector (Bruker AXS Advanced X-ray Solutions GmbH, Karlsruhe, Germany) were used to perform powder X-ray diffraction. The data were collected in the 10° to 90° 2θ range with a step of 0.03° 2θ and a counting rate of 57 s/step for the primary phase identification. The phase composition was identified using the ICSD database. XRD data were used to calculate crystallite size along the c-axis of the crystal structure of HA using the Bragg peaks (002) and corresponding Scherrer equation [19].

#### 2.4.3. Low-Temperature Absorption for Specific Surface Area Determination

The specific surface area and pore volume distribution of the powder samples were determined by low-temperature (77.4 K) nitrogen adsorption–desorption in a apparatus, NOVA 1200e (Quantachrome, Boynton Beach, FL, USA). The obtained isotherms were analyzed to calculate the specific surface area using the Brunauer–Emmett–Teller (BET) equation. The pore volume distribution was calculated according to the non-local density functional theory (NLDFT) method [20].

#### 2.4.4. Solid-State Nuclear Magnetic Resonance (NMR) Analysis

NMR spectra were recorded on a Bruker Avance HD III 600 NMR spectrometer operating at 599.90 MHz ^1^H frequency (242.84 MHz for ^31^P) using a 4 mm solid-state iProbe CPMAS DR-H&F VTN. The samples were loaded in 4 mm zirconia rotors and spun at a magic angle spinning (MAS) rate of 10 kHz for all measurements. The quantitative direct excitation ^31^P NMR spectra were recorded with a one-pulse sequence (Bruker Topspin library), 90° pulse length of 3.3 µs, 7 K time domain data points, spectrum width of 37 kHz, 32 scans, and a relaxation delay of 150 s. The spectra were processed with an exponential window function (line broadening factor 5) and zero-filled to 16 K data points. The ^1^H-^31^P cross-polarization magic angle spinning (CP MAS) spectra were acquired with the following experimental parameters: ^1^H excitation pulse of 2.5 μs, 3.5 ms contact time, 5 s relaxation delay, and 256 scans accumulated. An ^1^H SPINAL-64 decoupling scheme was used during the CP experiments. All ^31^P chemical shifts were referenced against the external solid reference NH_4_H_2_PO_4_ (δ 0.9 ppm). DMfit software (version: dmfit/x64/release #20220502) was used for the deconvolution, simulation, and fitting of the experimental NMR data [21].

## 3. Results

### 3.1. Synthesis of Hydroxyapatite

**Figure 1:**
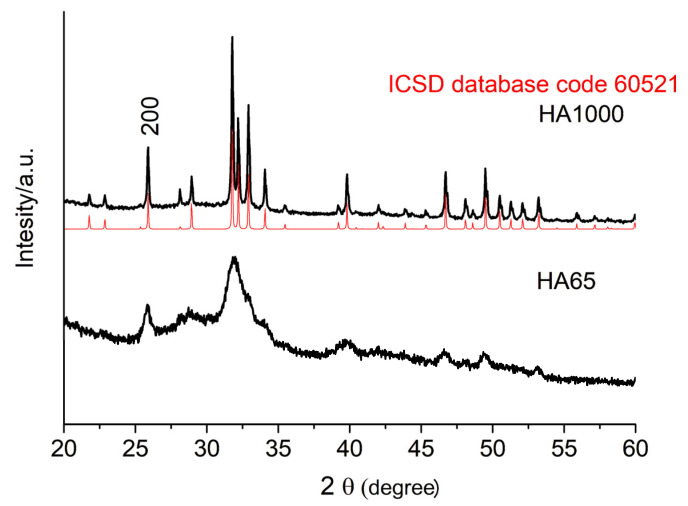
X-ray powder diffraction pattern of HA65 and HA1000 (black lines). The red line corresponds to calcium hydroxyapatite (Ca_5_(PO_4_)_3_OH) of ICSD database code 60521.

The results of the chemical analysis showed a ratio of Ca/P = 1.64 ± 0.01, close to the theoretical one for HA (Ca/P = 1.67 [18]). The X-ray diffraction pattern of HA65 (Figure 1) was characterized by broad peaks in the regions 24.9–26.7 2θ (degree) and 30.3–35.1 2θ (degree), which is typical of poorly crystalline substances with an apatite structure. The annealed sample (HA1000) was well-crystallized hydroxyapatite, the peaks of which corresponded to data from the ICSD X-ray database.

The crystallite size, calculated by the Scherrer equation using Bragg peaks (002) (Table 1), was in the size range of natural enamel and dentin, with the crystallites of sample HA65 being about seven times smaller than those of sample HA1000.

**Figure 2:**
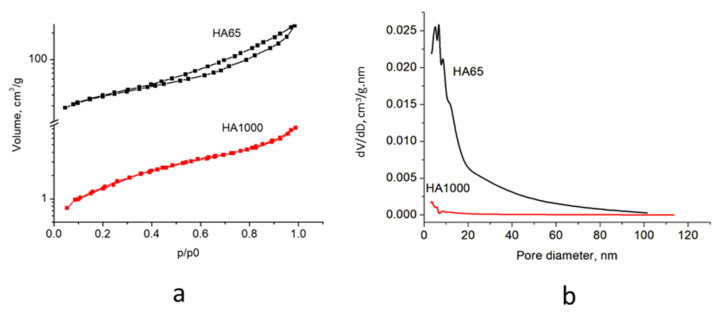
Low-temperature absorption characteristics of HA65 and HA1000: (**a**) adsorption–desorption isotherms and (**b**) pore size distribution.

The adsorption–desorption isotherms of the obtained materials are shown in Figure 2a, and the pore size distribution is displayed in Figure 2b. The specific surface areas (S_BET_), total pore volumes (V_t_), and average pore sizes (D_AV_) are summarized in Table 2.

**Table 1:** Crystallite size (in nm) calculated by the Scherrer equation with data from powder X-ray diffraction patterns.

Sample	Crystallite Size (Bragg Peaks (002))	Literature
HA65	20.2	This study
HA1000	141	This study
Enamel	89.7	[22]
	100	[23]
Dentin	30.9	[22]
	35	[23]

The studied samples show mixed type II–IV isotherms with H3-type hysteresis at p/p_0_ in the range of 0.41 to 1.0 (Figure 2a), indicating the formation of micro and mesopores [24]. The pore size distribution (Figure 2b) showed a significant difference in the porosity of the two materials. The porosity of HA65 was higher, with most of the most probable pore size in the range of 3.5–21 nm and a main peak centered at ~6.3 nm. The difference in pore size and crystallite size also determines a 23-fold higher specific surface area of HA65 compared to HA1000 (Table 2), but within the range of natural dentin and enamel.

**Table 2:** Specific surface area, volume, and average pore size of the obtained samples.

Sample	S_BET_, m^2^/g	V_t_, cm^3^/g	D_av_ nm
HA65	138	0.38	11
HA1000	6	0.01	8
Enamel [25]	8.7		
Dentin [25]	150		

**Figure 3:**
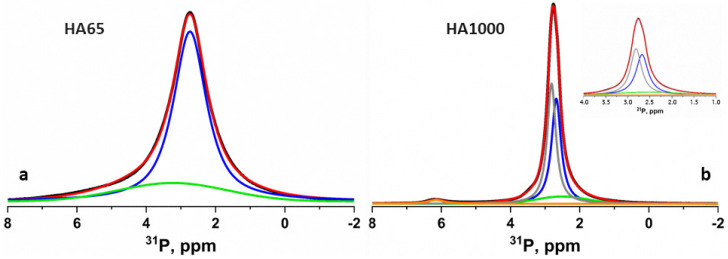
Direct excitation ^31^P NMR spectra of HA65 (**a**) and HA1000 (**b**). The experimental spectra are given in black, while the simulated spectra are presented in red lines. The individual contributions of the different components obtained after the deconvolution of the spectra are given with colored lines. The insert shows an expanded region around the main resonance in the spectrum of HA1000, where the two overlapping signals, with individual contributions given in blue and gray in the deconvoluted spectrum, are also visible.

The direct excitation ^31^P NMR spectra of HA65 and HA1000 are presented in Figure 3. The spectra of both samples show a characteristic resonance centered at around 2.8 ppm [26], typical for the hydroxyapatite phase. The overall linewidth of the resonance of HA65 is much broader compared to HA1000. The observed difference in the linewidths of the signals of the two samples could be explained by their different morphology. The broad spectral pattern of HA65 indicates the presence of a disordered amorphous HA phase. The smaller crystallites in HA65 compared to the size of the crystallites in HA1000 also result in the broadening of the resonance. To gain more detailed insight into the morphology of the two samples, the spectral patterns were further deconvoluted using DMFit software [21]. The deconvolution of the resonance of HA65 showed the presence of two components: the component corresponding to the intense narrow resonance at 2.8 ppm originated from a poorly crystalline HA, while the broad low-intensity component centered at around 3 ppm resulted from the highly disordered phase. The main resonance in the spectrum of HA1000 was composed of two narrow overlapping components at 2.7 and 2.9 ppm, corresponding to crystalline HA and Ca-deficient HA, respectively. A broad low-intensity signal centered at around 3 ppm was also observed, indicating the formation of a small amount of amorphous phase. The small resonance at around 6.2 ppm could be assigned to an unknown phase, possibly β-TCP formed as a result of the sintering at 1000 °C.

### 3.2. Interaction of the Functional Acidic Monomer 4-META with Hydroxyapatite

The changes in the solid phase and the interaction between the hydroxyapatite particles with the functional monomer 4-META were monitored by NMR spectroscopy using direct excitation ^31^P spectra, as well as ^1^H-^31^P and ^1^H-^13^C CPMAS spectra.

**Figure 4:**
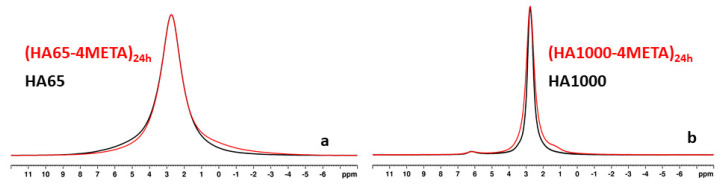
Direct excitation ^31^P NMR spectra: (**a**) HA65 and (HA65-4META)_24h_; (**b**) HA1000 and (HA1000-4META)_24h_.

The comparative analysis of the ^31^P NMR spectra of HA65 and those of the materials from the (HA65-4META) series showed that in all materials containing 4-META, there was a slight increase in the quantity of acidic phosphates, while the ratio of the amorphous to the nanocrystalline phase did not change significantly. Figure 4a shows the ^31^P spectra of HA65 (black line) and (HA65-4META)_24h_ (red line). The increased intensity of the broad shoulder in the range of 1.5 to −3.5 ppm typical for the acidic phosphate phases in the spectrum of (HA65-4META)_24h_ (red line) compared to the spectrum of HA65 (black line) in this region is clearly visible, while the overall linewidth at half height remains the same as for the HA65 resonance. The comparison of the ^31^P spectra of HA1000 with those of the (HA1000-4META) series (Figure 4b) shows that in materials containing 4-META, the signals are much broader, indicating a significant increase in the amount of the amorphous component. Additionally, in the sample with the longest contact time of 24 h (HA1000-4META)_24h_, an additional signal centered at 1.3 ppm is registered, suggesting the formation of dicalcium phosphate dihydrate (DCPD). This signal, visible in the spectrum of (HA1000-4META)_24h_, is likely also present in the spectrum of (HA65-4META)_24h_. However, due to overlap with the broad main resonance in the spectrum of (HA65-4META)_24h_, this weak signal cannot be observed on the ^31^P NMR spectrum illustrated in Figure 4a, but it appears as a broad asymmetrical shoulder at the base of the main signal in Figure 4a.

The increased amount of the acidic phases in the hybrid materials was further evidenced by the spectral patterns observed in the ^1^H-^31^P CP-MAS NMR spectra of the studied materials. In this technique, the resonances of the phosphorous species with H atoms in the vicinity of the P atom are selectively enhanced due to the transfer of magnetization from protons to the neighboring P nuclei. Figure S1 (See Supplementary Material) shows the ^1^H-^31^P CP MAS NMR spectra of pure HA65, pure HA1000, and the hybrid materials with the longest handling time ((HA65-4META)_24h_ and (HA1000-4META)_24h_). In the spectrum of (HA65-4META)_24h_, the presence of disordered hydrogen phosphate-containing phases is evidenced by the broad shoulder at around 0.5 ppm partially overlapped by the sharper peak at 2.9 ppm, assigned to the nanocrystalline HA phase. In the ^1^H-^31^P CP-MAS spectrum of (HA1000-4META)_24h_, the characteristic resonance for the DCPD at 1.3 ppm is narrow, indicating its crystalline nature. This signal is significantly enhanced compared to the HA resonance due to the more efficient magnetization transfer from the protons to P nuclei within the -HPO_4_^2−^ structural units of DCDP. The ^1^H-^31^P CP MAS NMR spectra of pure HA65 and HA1000 are dominated by the HA resonance, since they do not contain acidic phases.

**Figure 5:**
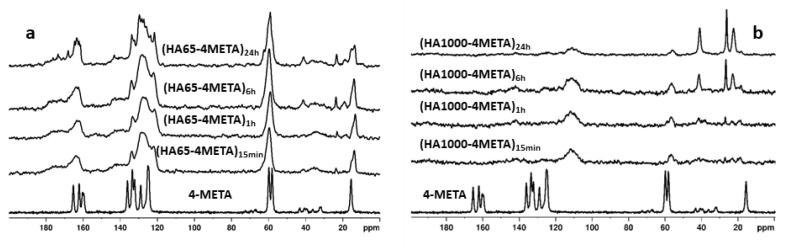
^1^H-^13^C CP MAS NMR spectra of the hybrid materials: (**a**) (HA65-4META) series; (**b**) (HA1000-4META) series compared to the spectrum of pure crystalline 4-META. The broad signal at 110 ppm in the spectra of HA1000-4META series is a background signal from the rotor KEL-F cap, which is visible due to the negligible intensity of the sample resonances.

Figure 5a,b, show the ^1^H-^13^C CP MAS NMR spectra of the HA65-4META and HA1000-4META sample series. For comparison, the spectrum of the pure 4-META monomer is also presented. The NMR spectrum of the pure monomer exhibits signals whose chemical shifts are characteristic of the carbon atoms in the different structural fragments of the molecule. The signals are narrow due to the crystalline nature of the monomer. In the spectra of the hybrid materials obtained from HA65 and 4-META, broad signals are observed, suggesting that in these materials, the 4-META component is in an amorphous state. As the contact time increases, both broad and narrow signals for 4-META appear in the spectra, indicating the formation of both amorphous and crystalline phases.

In the ^1^H-^13^C CP MAS NMR spectra of the hybrid samples obtained with HA1000, the signals of 4-META are hardly visible due to their very low intensity. The 20-fold lower specific surface area of HA1000 compared to HA65 is the reason for the insignificant adsorption of 4-META by the ceramic material. In addition, sintering at 1000 °C considerably reduced its reactivity and the possibility of interaction between Ca^2+^ ions and 4-META. With increasing contact time, new signals appear in the spectrum at 22, 27, and 45 ppm, indicating that additional processes such as hydrolysis or formation of short-chain methacrylic acid-based oligomers are taking place [27]. These additional resonances are also observed in the spectra of (HA65-4META) series at longer contact times, though with much lower intensity.

### 3.3. Stability of the Hybrids in Environments with Different pH

For these experiments, a sample of (HA65-4META)_24h_ was selected, which, according to NMR spectra (Figure 5a), contains the largest amount of 4-META. The time evolution of the aqueous phase pH and Ca^2+^ ions release was studied when the sample was immersed in two different liquid media: distilled water with pH 6.3 and 0.1 mol/L lactic acid solution with pH 4.5. For comparison, the behavior of pure HA65 was also investigated.

**Figure 6:**
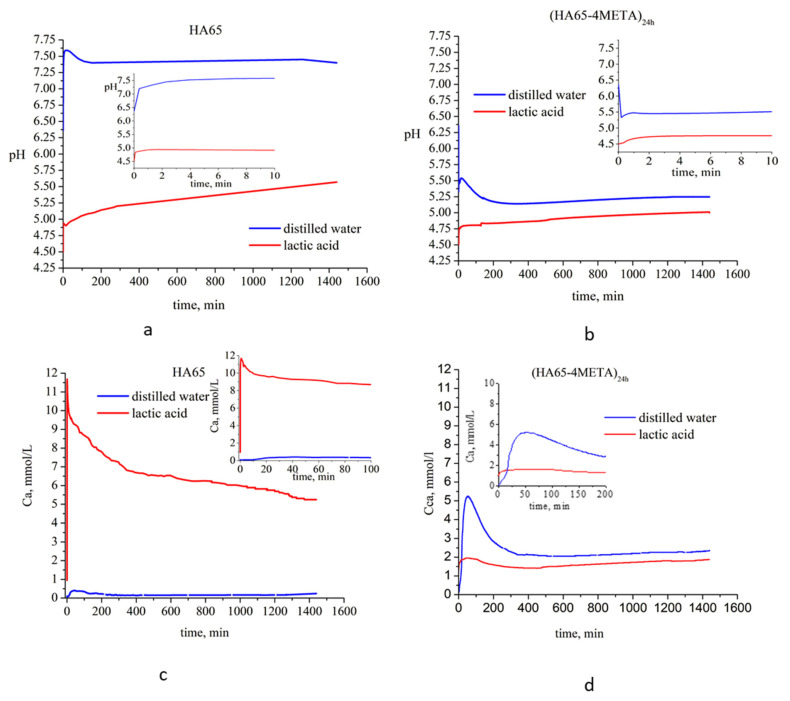
Kinetic profiles of pH (**a**,**b**) and concentration of free Ca^2+^ ions (**c**,**d**) measured in situ.

The results presented in Figure 6a demonstrate a steep increase in the pH of the aqueous phase after the first 5 min of sample immersion, reaching values of 7.6 and 4.9 in distilled water and lactic acid solution, respectively, when the solid sample used was solely HA65. For the (HA65-4META)_24h_ sample, the pH value surged abruptly to 4.76 in the lactic acid solution (Figure 6b). Conversely, when immersed in distilled water, the pH of the aqueous phase sharply decreased to 5.35. This is most likely due to the release of 4-META and its hydrolysis product into the water. Following minor fluctuations, the pH reached an almost constant value or displayed a low and smooth growth tendency.

The kinetic profiles of free Ca^2+^ ion content displayed similar trends: Ca^2+^ concentration in both aqueous phases and for both tested solid samples initially increased, then decreased until about the 6th hour, after which monotonic changes with time were observed (Figure 6c,d). The highest concentration of free Ca^2+^ ions was measured upon contact with HA65 with the lactic acid solution, and the lowest when HA65 was immersed in distilled water (Figure 6c). The concentration of free Ca^2+^ ions released upon contact of the (HA65-4META)_24h_ with the two liquids was found to be intermediate, but higher values were measured in water and lower in the lactic acid solution. Then, the Ca^2+^ concentrations in the two environments approached each other.

The measured total Ca concentrations after 14 days of immersion (Table 3) also showed the highest value (7.25 mmol/L) for the pure HA65 in 0.1 mol/L lactic acid, while the minimum value of 0.29 mmol/L was measured for the system HA65–distilled water. The total Ca^2+^ content after immersion of (HA65-4META)_24h_ in water and 0.1 mol/L lactic acid was almost the same—2.45 and 2.50 mmol/L, respectively.

**Table 3:** Total Ca and free Ca^2+^ ions after 14 days of immersion (mmol/L)

Solution	Free Ca^2+^ Ions	Total
HA65		
H_2_O	0.18 ± 0.01	0.29 ± 0.01
1 mmol/L lactic acid	5.24 ± 0.02	7.25 ± 0.01
(HA65-4-META)_24h_		
H_2_O	2.40 ± 0.01	2.45 ± 0.01
1 mmol/L lactic acid	1.50 ± 0.01	2.50 ± 0.01

The influence of the media on the solid phases after 14 days of immersion for the pure HA65 and the hybrid (HA65-4META)_24h_ in distilled water (H_2_O) and in lactic acid used (LA) was investigated by ^31^P NMR and ^1^H-^13^C CP MAS spectroscopy. The corresponding spectra are presented in Figures S2 and S3, respectively. Comparative analysis of the resonance linewidths in ^31^P spectra of the hybrid materials shows that the content of the amorphous component is highest in (HA65-4META)_24h_, while sample (HA65-4META)_24h_(LA) is characterized by the lowest amount of the amorphous phase. The changes in the morphology of HA65-based series of samples (Figure S2b) are less pronounced, with only the HA(LA) sample showing a minor decrease of the amorphous component. The ^1^H-^13^C CP MAS spectra of the hybrid materials (HA65-4META)_24h_, (HA65-4-META)_24h_(H_2_O) and (HA65-4META)_24h_(LA) demonstrate that after 14 days of immersion in distilled water and lactic acid solution, there is still a significant quantity of 4-META or its hydrolytic products present in the hybrid material (Figure S3).

## 4. Discussion

### 4.1. Interaction of the Functional Acidic Monomer 4-META with Hydroxyapatite

Hydroxyapatite (HA) is a naturally occurring mineral that serves as a key structural component of human bones and teeth. Its close chemical and structural similarity to the mineralized tissues in teeth—dentin and enamel—makes it an ideal model for studying their properties and developing dental applications. This study used synthetic hydroxyapatite to investigate its interaction with the functional monomer 4-methacryloxyethyl trimellitic anhydride (4-META) and the stability of the obtained hybrids in media of different pH.

The hydroxyapatite we synthesized after drying at 65° (HA65) exhibited a particle size (Table 1) and a specific surface area (Table 2) close to those of natural dentin. In addition, the low crystallinity of HA65 (Figure 1 and Figure 3b) and the presence of a large amount of amorphous phase (Figure 3a), the results of which are in accordance with the dentin study reported by Fujita-Nakajima et al. [28], inspired us to regard this material as an approximate model of dentin.

Hydroxyapatite obtained after sintering at 1000 °C (HA1000) has a particle size (Table 1) and specific surface area (Table 2) close to that of enamel. However, its significantly higher crystallinity (Figure 1 and Figure 3b), as well as the presence of a negligible amount of β-TCP (Figure 3b), define it as a ceramic material that is used as a restorative agent in dentistry.

The results of kinetic studies on the interaction of HA65 and HA1000 with 4-META showed that at the same immersion time in the freshly prepared solution of 4-META, the 4-META uptake was higher by dentin-like HA (HA65) than by HA ceramic (HA1000).

The ^1^H-^13^C CP-MAS NMR spectra of the two types of hybrid materials (HA64-4META) and (HA1000-4META) obtained with the two different hydroxyapatite powders show significant differences in their spectral patterns (Figure 5a,b). In the spectra of the (HA65-4META) series of samples, the resonances of 4-META are strong and their chemical shifts correspond to those of pure 4-META, while in the case of the (HA1000-4META) series, these signals are very weak at the level of the spectral noise. With increased contact time to 6 or more hours, the intensity of the new signals appearing at 22, 27, and 45 ppm increases in both materials. Figure 5a demonstrates that 4-META resonances in the hybrid HA65-4META materials are significantly broader compared to the resonances of the pure 4-META. The increased broadness of the peaks could be attributed to amorphization of the monomer or to possible polymerization of 4-META. However, we suggest that polymerization cannot be considered as the main reason for broadening of the resonances in the spectra of HA65-4META series of samples. In the case of polymerization, we would expect the appearance of additional resonances at around 45–50 ppm for the CH_2_ and quaternary C atoms from the main polymer chain. Nevertheless, this possibility has not been fully ruled out, particularly for samples (HA65-4META)_6h_ and (HA65-4META)_24h_, where the formation of a small number of polymeric structures can explain the appearance of the low-intensity resonances at 45 ppm from the CH_2_ and quaternary carbon atoms and at 22 and 27 ppm for the CH_3_ groups from the polymer structural units. On the other hand, previous studies demonstrated that in alcohol-based solutions, 4-META undergoes hydrolysis to 4-MET [14], with further esterification and degradation in several stages to different products such as methacrylic acid, 2-hydroxyethyl methacrylate, 4-(2-hydroxyethyl)-trimellitate, which are further hydrolyzed into ethylene glycol and trimellitic acid [27]. We suggest that some degradation products could be present in the HA65-4META materials; however, their signals would overlap with the broad 4-META resonances in the aromatic part of the spectrum, and only the resonances of the aliphatic moieties were visible within the region from 50 to 20 ppm, giving the additional resonances at 22, 27, and 45 ppm. However, the appearance of these resonances at longer contact times (6 h and 24 h) and their low intensity imply that the amount of the polymerized 4-META and/or the degradation products in HA65-based materials is very low. Therefore, we conclude that the main reason for broadening the resonances of 4-META in the HA65-4META series of materials is its amorphization in the presence of HA65.

Due to the highly amorphous nature of HA65 and its high specific surface area, the adsorption of 4-META from the alcohol–aqueous solution on HA65 particles is more efficient, resulting in stabilization of the monomer, thus preventing its fast hydrolysis and degradation in the alcohol–water solution during sample preparation. On the contrary, the adsorption of 4-META and its stabilization on HA1000 is not effective due to the significantly lower specific surface area, total pore volume, and pore size of HA1000. As a result, 4-META remains in the solution, where its degradation occurs with increased contact time, which can explain the lack of 4-META signals in the spectra of HA1000-4META materials and the appearance of the additional resonances in the region from 50 to 20 ppm in the spectrum originating from some degradation products that might be adsorbed on HA1000 particles.

Furthermore, the results reveal the occurrence of various processes leading to changes in the calcium phosphate phases under the conditions of the experiment. The appearance and accumulation of an acidic calcium phosphate phase, DCPD (Figure 4 and Figure S1), is observed over time, resulting from dissolution and crystallization processes in the acidic aqueous–alcoholic–4-META medium (pH 2).

### 4.2. Stability of the Hybrids in Environments with Different pH

The study of the stability of calcium phosphate–4META hybrids in various pH environments reveals their effectiveness and reliability in dental applications. This helps to predict their performance under real conditions. Acidic foods and beverages, oral bacteria, and metabolizing fermentable carbohydrates producing organic acids (e.g., lactic acid) can lower oral pH below 5, creating cariogenic conditions. It is therefore important to evaluate the behavior of materials used in adhesive systems under low-pH conditions.

For these studies, we chose pure HA65 and (HA65-4META)_24h_, due to the highest presence of 4-META in the latter. These samples served as approximate models of dentin and 4-META-treated dentin.

The kinetic studies on the stability of the (HA65-4META)_24h_ and HA65 in environments with different pH showed increased concentrations of free Ca^2+^ ions associated with the dissolution of the calcium phosphate phases (Figure 6c,d). Since calcium phosphates are poorly soluble substances, with solubility products in the order of 10^−6.5^ for CaHPO_4_·2H_2_O, 10^−25^ for the amorphous calcium phosphate to 10^−116.8^ for hydroxyapatite (Ca_10_(PO_4_)_6_(OH)_2_) [23], critical saturation and the solubility product of any of the calcium phosphate salts capable of precipitating from solution is reached very quickly. A reverse-precipitation process begins, in which the concentrations of free Ca^2+^ ions decrease until an equilibrium is reached between the dissolution and precipitation. In distilled water, critical saturation for pure substance (HA65) is achieved in about 30 min (Figure 6c), and for (HA65-4-META)_24h_ in 50 min (Figure 6d). The difference in the times to reach critical saturation and the differences in free Ca^2+^ concentrations is due to the medium pH and possible complexation of calcium in the solutions. The low content of free Ca ions upon contact of HA65 with water is due to the high pH of the solution (Figure 6a) and the lack of complexing agents that inhibit the solubility of HA (Figure 6c). When a sample (HA65-4META)_24h_ is immersed in water, the organic phase is released and acidic hydrolysis products of 4-META are formed, lowering pH (Figure 6b). Low pH stimulates dissolution, but complexation reactions of Ca^2+^ ions with organic components reduce the content of free Ca^2+^ ions. To reach critical saturation, it is necessary to dissolve a larger amount of solid phase, increasing solubility and extending the time (Figure 6d).

When dissolution is carried out in a lactic acid solution, critical saturation is reached faster—3 min at HA65 and 40 min at (HA65-4META)_24h_. The low initial pH of the solution (pH 4.5) promotes the dissolution of pureHA65 (Figure 6c), while additional complexation with the carboxylic group of lactic acid further reduces the concentration of free Ca^2+^ ions (Figure 6d).

The concentrations of free Ca^2+^ ions are lower than the total Ca concentration measured after 14 days (Table 3) for both samples due to the formation of the Ca complexes in the liquid phase.

The total Ca concentration upon dissolution of (HA65-4META)_24h_ in a 0.1 mol/L lactic acid solution is three times lower than the total Ca concentration upon dissolution of pure HA65. This, as well as the established presence of 4-META and its hydrolysis derivatives in the (HA65-4META)_24h_ after 14 days of immersion (Figure S3), indicates that the 4-META-treated dentin-like surfaces are more resistant to dissolution in an acidic environment than the untreated ones. The interaction between Ca^2+^ ions from the HA surface with the 4-META and its derivatives and their practical insolubility in water and water–salt solutions are responsible for the greater endurance of dentin-like HA treated with 4-META than pure HA.

## 5. Conclusions

Two types of hydroxyapatite with particle size, specific surface area, and crystallinity close to those of natural dentin (HA65) and hydroxyapatite ceramics (HA1000) were obtained.

It was found that dentin-like HA5 adsorbed 4-META in larger amounts than HA1000 ceramics when they immersed in an aqueous–alcoholic solution of 4-META. In both cases, the appearance of acid calcium phosphate over time was identified. However, the NMR studies showed that due to its favorable textural characteristics, HA65 efficiently prevented the hydrolysis and degradation of 4-META, while HA1000 4-META underwent changes under the experimental conditions, related to hydrolysis processes in the aqueous–alcoholic solution.

Upon contact of HA65 or its 4-META derivative with distilled water (pH 6.3) or lactic acid solution (pH 4.5), an initial dissolution process occurred, which was more intense in the lactic acid solution. After reaching a critical point, a reverse-crystallization process started. The time to reach critical saturation and the concentration of free Ca^2+^ ions in the solution depended on the properties of the liquids and especially on the complexation of Ca with the components of the liquid phase.

The total Ca^2+^ content in the solution after 14 days of immersion in the lactic acid solution showed that both pure HA and the hybrid dissolved over time, but the solubility of the hybrid was three times lower. This suggests that the dentin-like surfaces treated with 4-META exhibit greater resistance to dissolution in an acidic environment compared to the untreated surfaces.

## Data Availability

The original contributions presented in this study are included in the article/Supplementary Material. Further inquiries can be directed to the corresponding authors.

## Supplementary Materials

The following supporting information can be downloaded at https://www.mdpi.com/article/10.3390/ma18081689/s1. Figure S1. ^1^H-^31^P CP-MAS NMR spectra of pure HA65, pure HA1000 and the hybrid materials obtained at the longest contact time of 24 h, (HA65-4META)_24h_ and (HA1000-4META)_24h_. (please see the main text for detailed explanation of the spectra); Figure S2. ^31^P ЯMP spectra of: (a) (HA65-4META)_24h_—black line; (HA65-4META)_24h_(H_2_O)—blue line and (HA65-4-META)_24h_(LA)—red line and (b): HA65—black line; HA65(H_2_O)—blue line and HA65(LA)—red line; Figure S3. ^1^H-^13^C CP-MAS NMR spectra of pure (HA65-4META)_24h_, (HA65-4META)_24h_(H_2_O) and (HA65-4META)_24h_(LA).
