# Supplementary material for: Studies on the Interaction Between the Functional Monomer 4-Methacryloxyethyl Trimellitic Anhydride and Hydroxyapatite and Stability of the Obtained Hybrids
Source: Materials (Basel). 2025 Apr 8;18(8):1689. doi: 10.3390/ma18081689 (PMC12029120; doi:10.3390/ma18081689)
Supplement: Supplementary file 1 [file materials-18-01689-s001.zip › materials-3487877-supplementary.pdf]

# Studies on the Interaction Between the Functional Monomer 4-Methacryloxyethyl Trimellitic Anhydride and Hydroxyapatite and Stability of the Obtained Hybrids

**Vasil Kalchinov [1,*], Kostadinka Sezanova [2], Pavletta Shestakova [3], Sevda Yantcheva [1], Radosveta Vasileva [1] and Diana Rabadjieva [2,*]**

## Supplementary Material

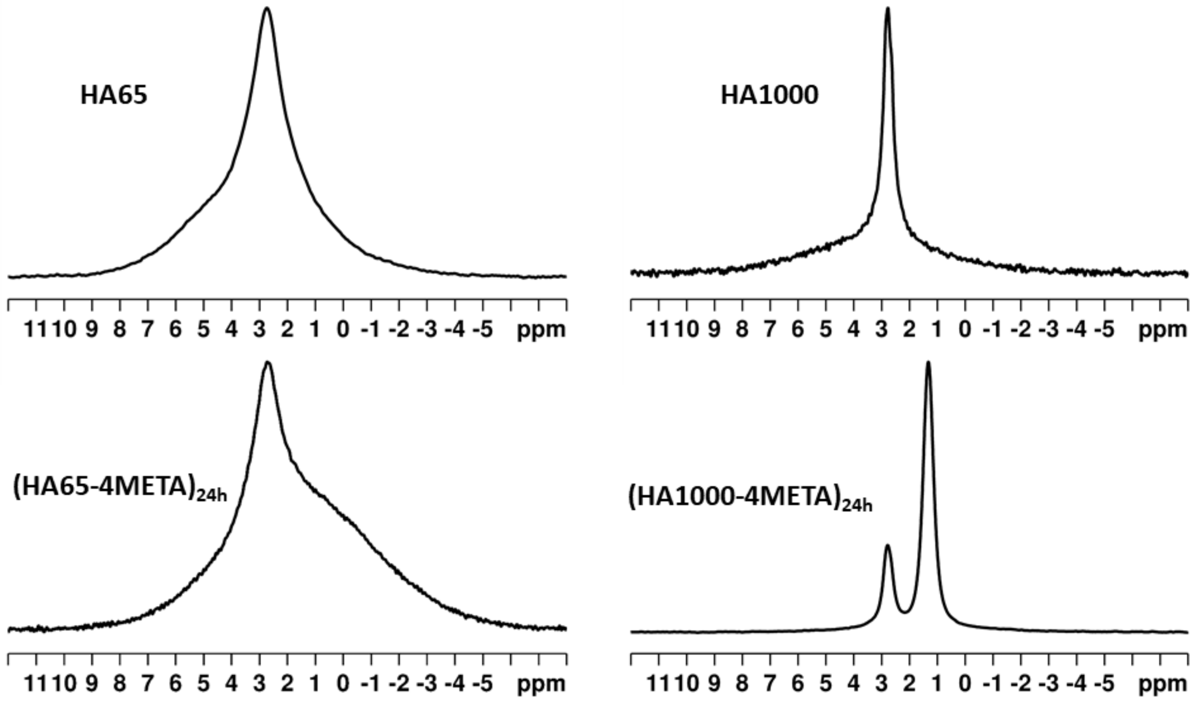


Figure S1. $^{1}H$-$^{31}P$ CP-MAS NMR spectra of pure HA65, pure HA1000 and the hybrid materials obtained at the longest contact time of 24h, $(HA65\text{-}4META)_{24h}$ and $(HA1000\text{-}4META)_{24h}$. (please see the main text for detailed explanation of the spectra).

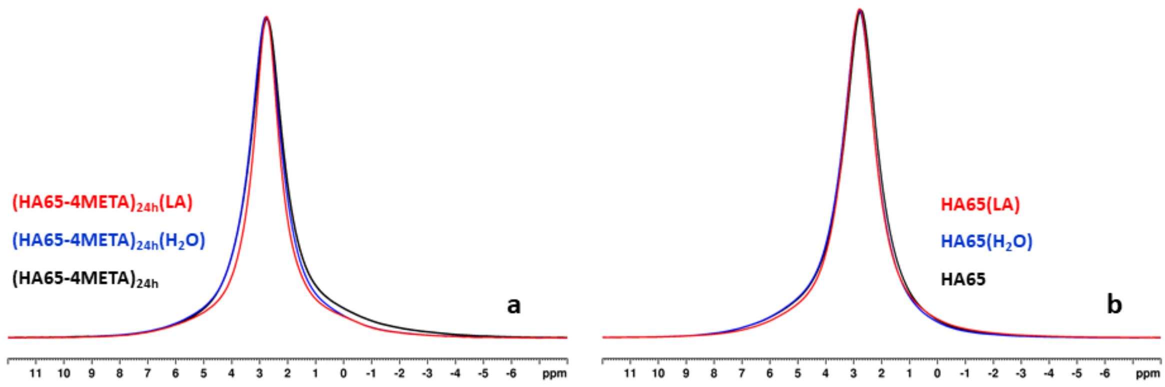


Figure S2. $^{31}$P ЯМР spectra of: (a) (HA65-4META)$_{24h}$ – black line; (HA65-4META)$_{24h}$($H_2O$) – blue line and (HA65-4-META)$_{24h}$(LA)- red line and (b): HA65 – black line; HA65($H_2O$)– blue line and HA65(LA) – red line.

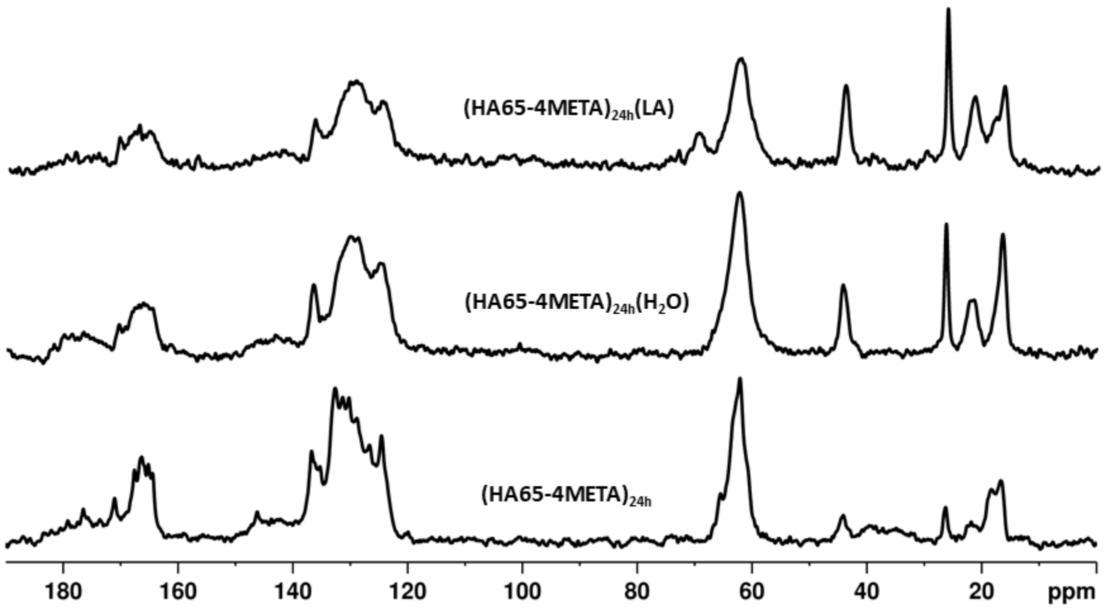


Figure S3. $^1$H-$^{13}$C CP-MAS NMR spectra of pure (HA65-4META)$_{24h}$, (HA65-4META)$_{24h}$($H_2O$)and (HA65-4META)$_{24h}$(LA).
